# Non-Dispersive Infrared Sensor for Online Condition Monitoring of Gearbox Oil

**DOI:** 10.3390/s17020399

**Published:** 2017-02-18

**Authors:** Markus S. Rauscher, Anton J. Tremmel, Michael Schardt, Alexander W. Koch

**Affiliations:** Institute for Measurement Systems and Sensor Technology, Technical University of Munich, 80333 Munich, Germany; a.tremmel@tum.de (A.J.T.); m.schardt@tum.de (M.S.); a.w.koch@tum.de (A.W.K.)

**Keywords:** oil condition monitoring, gearbox lubricant, non-dispersive infrared, oil oxidation, water content, acid number

## Abstract

The condition of lubricating oil used in automotive and industrial gearboxes must be controlled in order to guarantee optimum performance and prevent damage to machinery parts. In normal practice, this is done by regular oil change intervals and routine laboratory analysis, both of which involve considerable operating costs. In this paper, we present a compact and robust optical sensor that can be installed in the lubrication circuit to provide quasi-continuous information about the condition of the oil. The measuring principle is based on non-dispersive infrared spectroscopy. The implemented sensor setup consists of an optical measurement cell, two thin-film infrared emitters, and two four-channel pyroelectric detectors equipped with optical bandpass filters. We present a method based on multivariate partial least squares regression to select appropriate optical bandpass filters for monitoring the oxidation, water content, and acid number of the oil. We perform a ray tracing analysis to analyze and correct the influence of the light path in the optical setup on the optical parameters of the bandpass filters. The measurement values acquired with the sensor for three different gearbox oil types show high correlation with laboratory reference data for the oxidation, water content, and acid number. The presented sensor can thus be a useful supplementary tool for the online condition monitoring of lubricants when integrated into a gearbox oil circuit.

## 1. Introduction

Gearbox oils are exposed to high mechanical loads, wide ranges of temperature, and the possibility of contamination with liquid or solid impurities. As these operating conditions lead to the degradation of the oil, it is exchanged at regular intervals in most applications to prevent damage to the machinery. The time between changes is usually chosen conservatively, which leads to unnecessary material and labour costs and increases environmental pollution.

For large machinery with high oil volumes, regular laboratory analysis of the oil is carried out. Although this technique allows oil change intervals to be realized according to the actual requirements, additional costs arise from sample drawing and analysis. Especially in remote facilities like offshore wind turbines, regularly drawing samples requires high effort. Additionally, sudden deterioration of the oil—e.g., contamination with cooling water from a leaking seal—remains undetected, and can cause severe damage over a short period of time.

Online sensors that are directly installed at a facility and which continuously monitor the oil condition may present a solution to this complex of problems. Different approaches have been made in this field, based on impedance spectroscopy [1,2], oil debris sensors [3,4], or viscosity measurement [5], for example. An overview of sensors currently used for the monitoring of wind turbine gearbox oil is given in [6], and the sensors are tested under different environmental conditions. Infrared (IR) spectroscopic principles have proven successful in determining different parameters associated with oil degradation, thus creating a more complete prediction of the oil condition [7,8,9]. As infrared spectrometers are relatively expensive and are usually not robust enough to be operated in industrial environments, approaches that use non-dispersive infrared (NDIR) measurement setups have been made to monitor additive depletion [10] or oil oxidation [11]. NDIR setups for monitoring phosphate-ester hydraulic fluid [12,13], gas engine oil [14], and diesel engine oil [15] have been presented.

However, these oil types differ from gearbox oils with respect to the chemical properties of the base stock and additive composition. Moreover, the operating conditions, expected contaminants, and relevant condition parameters are different for gearbox applications. In this paper, we present a working NDIR sensor for the measurement of three gearbox oil condition parameters: oxidation, water content, and acid number (AN). Starting from a general analysis of the reasons for oil deterioration, we investigate the impact of oil aging on the infrared absorption spectrum of gearbox oils. We introduce a method based on multivariate partial least squares regression to select spectral regions suitable for monitoring the AN. Based on the findings of this regression analysis, we select optical bandpass filters to be implemented in the sensor prototype. The optical design of the sensor prototype is simulated with a ray tracing software. The presented sensor prototype is realized in a compact and rugged aluminum housing appropriate for use in industrial environments.

## 2. Gear Oil Degradation

Demanding operating conditions lead to a degradation of the gearbox oil used in industrial applications. Laboratory oil analysis examines different oil condition parameters to monitor the level of degradation and to estimate the remaining service life. Three such parameters that are relevant for gearbox oil are oxidation, water content, and acid number. This section gives an overview of the causes and consequences of the degradation processes, as well as common monitoring methods.

### 2.1. Oxidation

The agitation experienced by the oil in a gearbox leads to increased contact between the oil and oxygen from the air. In conjunction with high temperatures, the oil can react with oxygen molecules, altering its chemical and physical properties. As a consequence, the viscosity of the oil increases. Further polymerization of the oxidation products can cause varnish, sludge, and hard compounds to form, which diminish the oil flow [16]. A detailed description of the oxidation process that considers the influence of temperature and catalytic reactions can be found in [17].

In laboratory practice, the oxidation of used lubricants is measured by infrared spectroscopy according to ASTM E2412 [18].

### 2.2. Water Content

Increased water content in the oil can occur because of leakage, pressure washing, improper storage conditions for fresh oil, or condensation in the gearbox housing. Too much water reduces the load-bearing ability of the oil, which leads to increased wear on highly-stressed surfaces. In addition, water promotes the oxidation process of the oil, causes the machinery parts to corrode, and may lead to the sedimentation of additives [19].

In laboratory practice, the water content of used oils is often measured by Karl–Fischer titration according to ASTM D6304 [20]. Additionally, ASTM E2412 describes a method of using infrared spectroscopy to quantify the water content in used oil [18].

### 2.3. Acid Number

The acid number (AN) describes the mass of potassium hydroxide (KOH) in milligrams that is needed to neutralize the acids contained in one gram of used oil. The AN is therefore a measure of the amount of acidic compounds in the oil, and is thus closely related to the risk of corrosion of machinery parts. As many different sources of oil degradation affect the AN, it has been established as a key parameter in oil condition monitoring.

In laboratory analysis, the AN is measured by titrating the used oil sample with KOH as described in ASTM D664 [21]. To increase repeatability and throughput, many laboratories use modified measurement methods whose results cannot be directly compared to the standardized practice.

## 3. Oil Condition Monitoring by Means of Infrared Spectroscopy

As described in Section 2, the deterioration and contamination of oil is accompanied by a change in the concentration of its chemical compounds. Infrared spectroscopy can be used to monitor the concentration of different chemical compounds, and thus the oil degradation process. The principle of infrared spectroscopy is based on the fact that chemical compounds absorb infrared light with different strengths depending on the wavenumber $\tilde{\nu }$. According to the Lambert–Beer law stated in Equation (1), the absorption spectrum $A(\tilde{\nu })$ depends linearly on the molar attenuation coefficient $\alpha (\tilde{\nu })$ of the compound, its concentration *c*, and the length of the path *d* that the light takes through the material [22].

(1) $$A\left(\tilde{\nu }\right)=c\;d\;\alpha \left(\tilde{\nu }\right)$$

The absorption spectrum of a sample is calculated from its transmission spectrum $T(\tilde{\nu })$ using the decadic logarithm according to Equation (2). Here, $I_{1}\left(\tilde{\nu }\right)$ is the intensity measured when the sample is placed in a beam of infrared light, and $I_{0}\left(\tilde{\nu }\right)$ is the intensity measured without the sample.

(2) $$A\left(\tilde{\nu }\right)=-\log_{10}\left(T\left(\tilde{\nu }\right)\right)=-\log_{10}\left(\frac{I_{1}\left(\tilde{\nu }\right)}{I_{0}\left(\tilde{\nu }\right)}\right)$$

In the following, we analyze the infrared absorption spectra of used gearbox oil samples that were measured with a Fourier transform infrared spectrometer. Based on this analysis, we select spectral ranges suitable for monitoring the oxidation, water content, and acid number of the oil.

### 3.1. Oxidation

**Figure 1:**
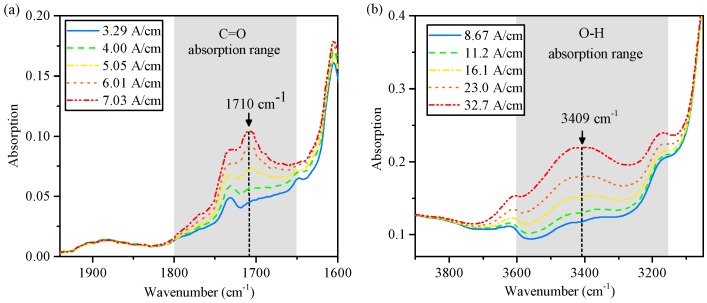
(**a**) Section of the infrared absorption spectrum of a wind turbine gearbox oil with different oxidation levels ranging from 3.29 A/cm to 7.03 A/cm according to ASTM E2412; (**b**) Section of the infrared absorption spectrum of an automotive gearbox oil contaminated with different concentrations of water ranging from 0 ppm to 1991 ppm, corresponding to ASTM E2412 readings of 8.67 A/cm to 32.7 A/cm.

Figure 1a shows a section of the absorption spectrum of a wind turbine gearbox oil (WTO) in different stages of aging. The spectra show that the infrared absorption of the oil increases in the spectral range from 1800 cm${}^{-1}$ to 1650 cm${}^{-1}$, exhibiting a peak at 1710 cm${}^{-1}$. The change in the infrared spectrum is due to the higher concentration of carbonyl groups that show strong absorption in this region [23]. Because this functional group is contained in many oxidation by-products, the infrared absorption in this spectral range can be used as a measure of oil oxidation. For the oil samples depicted in Figure 1a, the level of oxidation was calculated according to ASTM E2412, and ranges from 3.29 absorption units per cm (A/cm) to 7.03 A/cm. To monitor the change in the infrared absorption around 1710 cm${}^{-1}$, a suitable optical bandpass filter is selected in the context of the NDIR sensor implementation described in Section 4.

### 3.2. Water Content

Figure 1b shows a section of the absorption spectrum of an automotive gearbox oil (AGO) that was dispersed with different concentrations of distilled water ranging from 0 parts per million (ppm) to 1991 ppm. To measure the water content, the spectral absorption of the hydrogen-bonded hydroxy stretch vibration is used, exhibiting a broad absorption band from 3600 cm${}^{-1}$ to 3150 cm${}^{-1}$ with a peak at 3409 cm${}^{-1}$. Measuring the water content according to the method described by ASTM E2412 results in readings of 8.7 A/cm to 32.7 A/cm for the spectra depicted in Figure 1b. To monitor the change in the infrared absorption around 3409 cm${}^{-1}$, a suitable optical bandpass filter is selected in context of the NDIR sensor implementation described in Section 4.

### 3.3. Acid Number

Other than oxidation products and water content, many different chemical compounds influence the acid number of oil. Intermediate and final products of the oxidation process, carboxylic and organic acids and additives or their decomposition products can react with the titration solution, and therefore influence the value of the AN [16]. Consequently, a change in the AN affects multiple regions in the absorption spectrum.

We use multivariate partial least squares (PLS) regression to analyze the relation between the infrared absorption spectrum and the AN of used oil samples. The PLS algorithm constructs a regression model consisting of a scalar $b_{0}$ and a vector $b_{1}$ that describes the relation between a set of independent variables *x* and a response variable *y* as described by Equation (3). In our case, *x* is the infrared absorption spectrum and *y* is the AN of an oil sample. The values of $b_{0}$ and $b_{1}$ are optimized by the PLS algorithm in order to minimize the residual *ϵ*.

(3) $$y=b_{0}+xb_{1}+\epsilon$$

The regression coefficient vector $b_{1}$ gives a measure for the influence of the infrared absorption on the AN at different wavenumbers [24]. Thus, wavenumbers where $b_{1}$ exhibits a maximum denote spectral regions that strongly influence the AN.

We performed PLS regression analysis on a data set consisting of infrared absorption spectra and the related AN of used wind turbine oil (WTO) and marine gearbox oil (MGO) samples. Table 1 gives a summary of the data set of oil samples at our disposal. All three oil types are mineral industrial gearbox oils.

**Figure 2:**
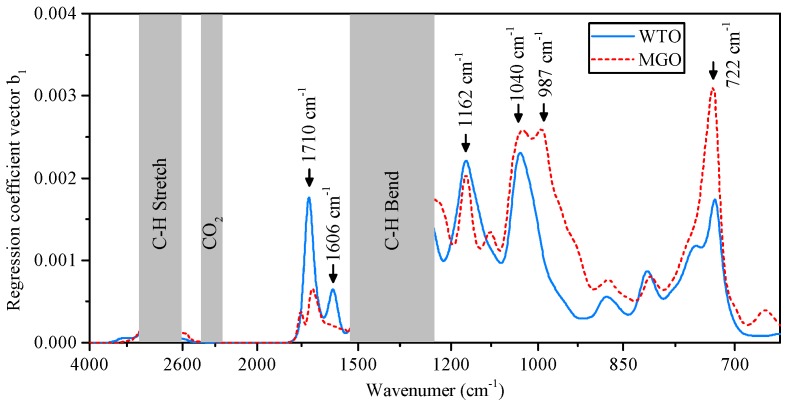
Regression coefficient vector $b_{1}$ constructed by the partial least squares (PLS) algorithm for the WTO and the MGO oil samples. The local maxima of $b_{1}$ are highlighted, as they denote spectral regions with high influence on the acid number (AN).

Figure 2 shows the regression coefficient vector $b_{1}$ for the WTO and the MGO. For the regression analysis, the absorption spectra were filtered with a Gaussian filter of 2% bandwidth to simulate the decreased spectral resolution resulting from the use of optical bandpass filters in the NDIR sensor. The regions of strong C-H absorption from 3100 cm${}^{-1}$ to 2700 cm${}^{-1}$ and 1540 cm${}^{-1}$ to 1250 cm${}^{-1}$ were excluded from the regression analysis due to the non-linearity of the infrared absorption in these spectral ranges, as well as the CO_2_ absorption band from 2400 cm${}^{-1}$ to 2300 cm${}^{-1}$.

The most prominent maxima of $b_{1}$ are highlighted in Figure 2 and labeled with the corresponding wavenumbers. To monitor the change in infrared absorption in these spectral regions of high influence on the AN, suitable optical bandpass filters are selected in the context of the NDIR sensor implementation described in Section 4.

### 3.4. Baseline Correction

As small particles in the oil lead to scattering of the infrared light, higher absorption values are measured as the particle count increases. To accommodate for this fact, a baseline correction of the absorption value must be carried out. Because the scattering has a higher impact on shorter wavelengths and thus induces a sloping baseline, two baseline points at 3900 cm${}^{-1}$ and 2100 cm${}^{-1}$ are established to linearly compensate for this effect. In these spectral regions, none of the typical contaminants or degradation products of the oil aging process have a significant influence on the absorption spectrum. To apply a baseline correction to the absorption values measured with the presented NDIR sensor, suitable optical bandpass filters are selected in the context of the implementation process described in Section 4.

## 4. Sensor Implementation

**Figure 3:**
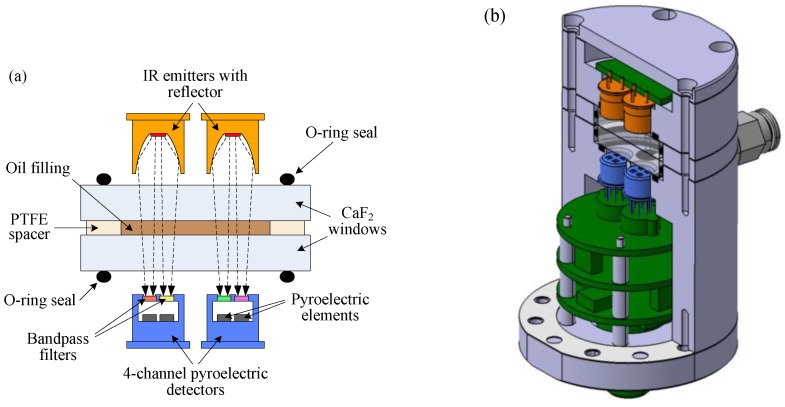
(**a**) Functional principle of the sensor implementation. The light emitted by two infrared (IR) emitters travels through the measurement cell containing the oil sample being tested. It is then detected by two four-channel pyroelectric detectors equipped with different optical bandpass filters; (**b**) Sectional computer-aided design model of the sensor prototype.

Figure 3a shows the functional principle of the presented NDIR oil condition sensor. The measurement cell consists of two calcium fluoride (CaF_2_) windows with a diameter of 25.4 mm and a thickness of 5 mm, separated by a polytetrafluoroethylene (PTFE) spacer of 0.1 mm thickness. One of the windows is drilled to provide a hydraulic connection to the lubrication circuit. In order to achieve sufficient pressure resistance, the windows are compressed with o-ring seals on either side when the cell is installed in the sensor housing. Two thin-film hot plate infrared sources with reflectors (Micro-Hybrid JSIR350) emit radiation in the mid-infrared range that travels through the windows and the oil filling between them. Depending on the condition of the oil, the light is absorbed to different extents at different wavenumbers. The transmitted light is then collected by two four-channel pyroelectric detectors (Micro-Hybrid PS4x2C1). The detector channels are fitted with optical thin-film bandpass filters to monitor different regions of the infrared absorption spectrum of the oil. Figure 3b shows a detailed drawing of the sensor mechanical design featuring the internal optics and electronics.

**Figure 4:**
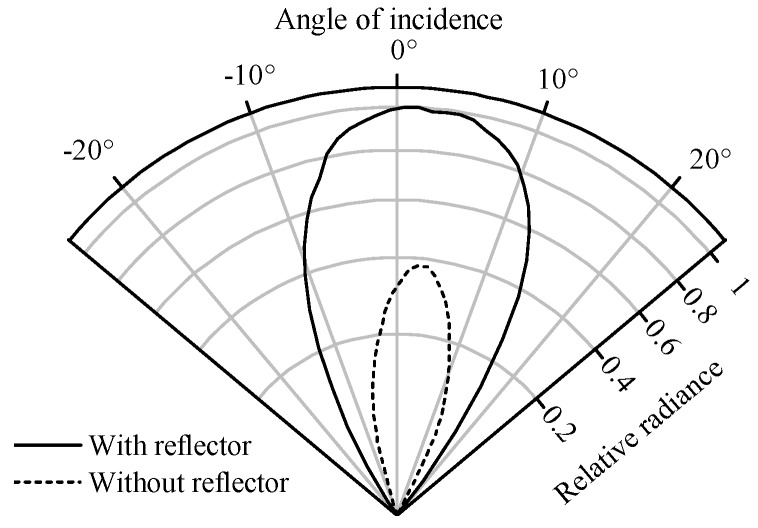
Simulation result showing the angular distribution of the light passing through the bandpass filters for emitters with and without reflectors.

When selecting optical bandpass filters to monitor the spectral ranges identified in Section 3, the angle of the light passing through the bandpass filters must be considered. This is because the wavenumber of maximum transmission of optical thin-film interference filters increases as the angle of incidence increases. To estimate the influence of this effect in our application, we performed a ray tracing analysis of the optical setup using the software ZEMAX. Figure 4 shows the simulated angular distribution of the radiance passing through the bandpass filters when using emitters with and without a reflector. An emitter with a reflector was used in the presented setup, because the total power received by the detector is 2.5 times higher than when an emitter is used without a reflector. However, as seen in Figure 4, the maximum angle of incidence on the bandpass filters increases to 20${}^{\circ }$ when an emitter with a reflector is used, compared to 13${}^{\circ }$ when an emitter without a reflector is used.

To estimate the wavenumber of maximum transmission of thin-film bandpass filters when inserted in this optical setup, we use the approach described in [25]. Equation (4) gives the relation between the wavenumber of maximum transmission ${\tilde{\nu }}_{⊥}$ at normal incidence and the wavenumber of maximum transmission ${\tilde{\nu }}_{\Theta }$ of the filter for cone-shaped incident light. Here, Θ is the semi-cone angle of the incident light and $n^{*}$ is the effective refractive index of the filter.

(4) $${\tilde{\nu }}_{⊥}={\tilde{\nu }}_{\Theta }{\left(1+\frac{\Theta^{2}}{4{n^{*}}^{2}}\right)}^{-1}$$

The first column of Table 2 gives an overview of the spectral features that must be monitored in order to derive the oxidation, water content, and acid number of the oil as described in Section 3. The second column of Table 2 gives the spectral position of these features as derived from the findings displayed in Figure 1 and Figure 2. For best performance, the wavenumbers of maximum transmission ${\tilde{\nu }}_{\Theta }$ of the bandpass filters should preferably be located at these spectral positions when they are inserted into the presented optical setup. As the parameters of optical bandpass filters are usually specified for normal incident light, we use Equation (4) to calculate the target value of ${\tilde{\nu }}_{⊥}$ from the target value of ${\tilde{\nu }}_{\Theta }$, as stated in the third column of Table 2.

**Figure 5:**
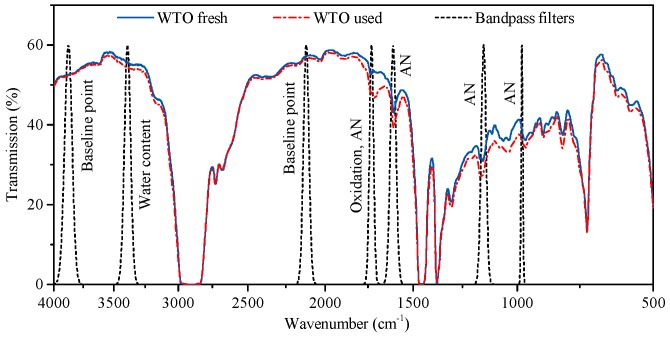
Transmission spectrum of one fresh and one used WTO sample and illustration of the optical bandpass filters selected for the non-dispersive infrared (NDIR) sensor implementation.

To ensure that the presented NDIR oil condition sensor remains economically feasible, we selected commercially available optical bandpass filters with parameters matching the targeted ${\tilde{\nu }}_{⊥}$ as closely as possible. The fourth column of Table 2 gives the parameters of the optical bandpass filters used for the sensor implementation. As most of the employed bandpass filters yield a full width at half maximum (FWHM) of about 2% of $v_{⊥}$, reasonable overlap of the bandpass filter transmittance range and the targeted spectral ranges is achieved. However, no commercial bandpass filters to monitor the spectral ranges around 1040 cm${}^{-1}$ and 722 cm${}^{-1}$ were available. The remaining seven bandpass filters stated in Table 2 were fitted into the housing of the pyroelectric detectors as depicted in Figure 3a. Finally, Figure 5 illustrates the transmittance of all bandpass filters implemented in the presented NDIR sensor and shows the transmission spectrum of one fresh and one used WTO sample.

**Figure 6:**
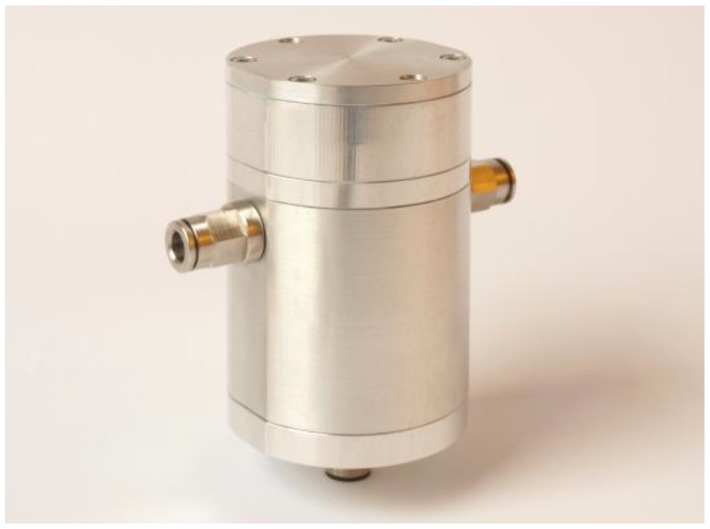
Assembled NDIR oil sensor prototype featuring 6 mm push-in hose connectors on either side and an electrical connector at the bottom of the sensor housing.

All optical and electronic components of the sensor were designed to fit in a rugged aluminum housing with a diameter of 55 mm and a length of 82 mm. Figure 6 shows a picture of the working sensor prototype. A pressure of 0.8 MPa (116 psi) was applied to the measuring cell for three days with no signs of leakage.

## 5. Data Processing and Sensor Calibration

To measure the absorption *A* of an oil sample in each channel of the NDIR sensor, a background measurement of $I_{0}$ without a sample must be carried out first. To avoid interference effects introduced by the empty measurement cell, $I_{0}$ is measured in the optical setup without the CaF_2_ windows of the measurement cell installed. The reinstalled measurement cell is then filled with oil, the measurement of $I_{1}$ is taken, and the absorption is calculated for each channel according to Equation (2).

**Figure 7:**
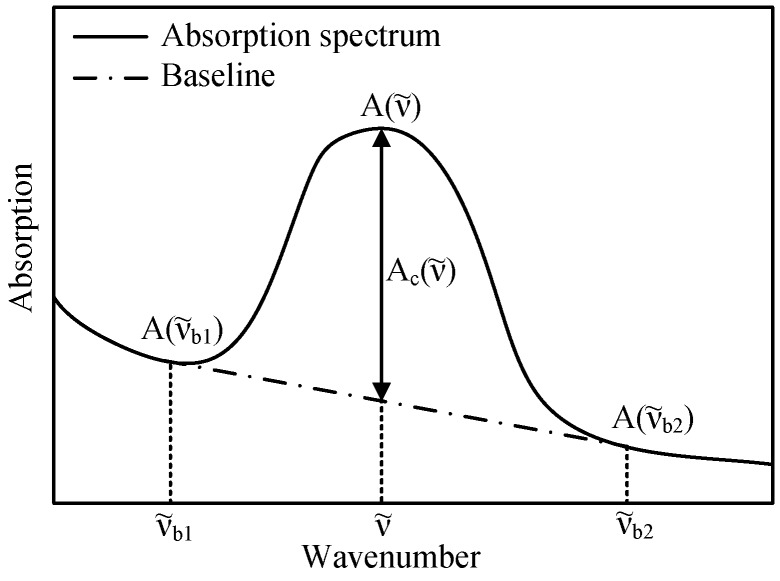
Functioning principle of the method used to correct for the sloping baseline of the infrared absorption spectrum.

To compensate for the sloping behaviour of the infrared absorption spectrum, the baseline of the measured absorption values is corrected as represented in Figure 7. Equation (5) is used to calculate the baseline-corrected absorption value $A_{c}$ for each channel. The wavenumbers ${\tilde{\nu }}_{b1}$ and ${\tilde{\nu }}_{b2}$ describe the bandpass filters used for baseline correction as stated in Table 2.

(5) $$A_{c}\left(\tilde{\nu }\right)=A\left(\tilde{\nu }\right)-A\left({\tilde{\nu }}_{b1}\right)-\frac{A\left({\tilde{\nu }}_{b1}\right)-A\left({\tilde{\nu }}_{b2}\right)}{{\tilde{\nu }}_{b1}-{\tilde{\nu }}_{b2}}\left(\tilde{\nu }-{\tilde{\nu }}_{b1}\right)$$

A calibration model is used to derive the oxidation, water content, and acid number from the infrared absorption measured with the NDIR sensor. We use multivariate PLS regression to construct a linear calibration model according to Equation (3). In this case, *x* is a vector containing the baseline-corrected absorption values $A_{c}$ of all channels, and *y* is the condition parameter reference value of the oil sample. The reference values for the oil condition parameters of the samples stated in Table 1 were generated according to appropriate standards. For the AN, the reference values were measured in a laboratory according to ASTM E664. The reference values for the oxidation and the water content were calculated from the infrared absorption spectrum according to ASTM E2412. As the infrared absorption behaviour differs between oil types, the regression models are only valid for the oil with which they were constructed.

## 6. Measurement Results and Discussion

All samples of the data set stated in Table 1 were measured with the sensor prototype. The samples were injected into the measuring cell with a syringe, and the cell was flushed with petroleum benzine 40–60 between two samples. Readings from the two detectors were taken consecutively to avoid diagonal irradiation from the emitters to the detectors, and thus large angles of incidence that would alter the transmission behaviour of the bandpass filters.

**Figure 8:**
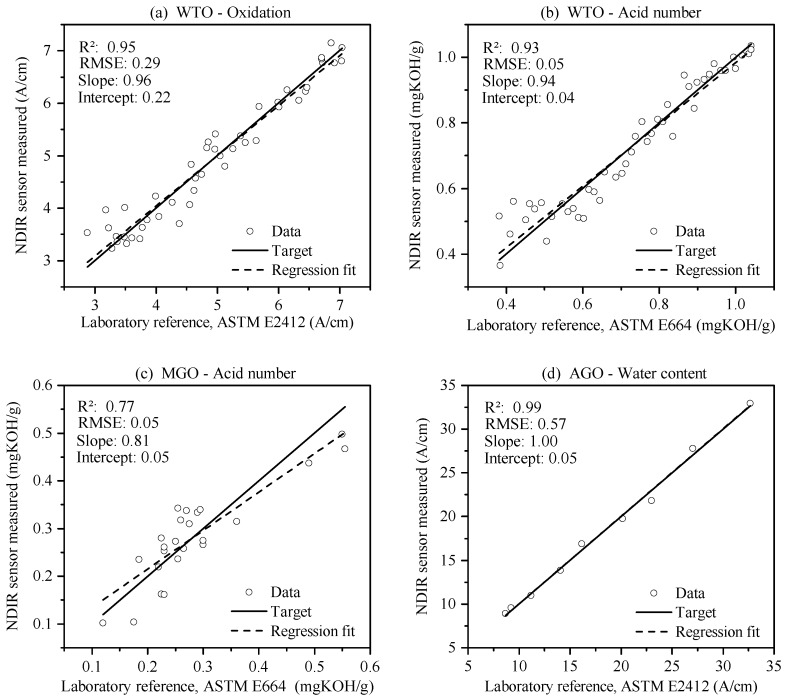
(**a**) Model validation for the oxidation measurement of the WTO samples; (**b**) Model validation for the AN measurement of the WTO samples; (**c**) Model validation for the AN measurement of the MGO samples; (**d**) Model validation for the water content measurement of the AGO samples. RMSE: root mean square error.

We used leave-one-out cross-validation to construct and validate the calibration models for the sensor, as this method has been proven to perform well on small data sets [26]. Figure 8 shows the results of the sensor cross-validation process for some condition parameters in the form of a reference-vs.-measured plot. The horizontal axis shows the reference value of the samples, while the vertical axis shows the corresponding value measured with the NDIR sensor using the appropriate calibration model.

Table 3 shows the coefficient of determination R${}^{2}$, the root mean square error (RMSE), the slope, and the intercept for all condition parameters of each oil type. For the water contamination, the model performance regarding R${}^{2}$, slope, and intercept for the AGO is superior to the WTO and MGO. However, the RMSE is lower for the WTO and MGO. We attribute this behaviour to the fact that the WTO and MGO samples show only a low level of water contamination compared to the AGO samples, which results in a minor change in the infrared absorption values, and thus a higher noise-related error in the calibration model.

In addition, the MGO performs more poorly in the prediction of oxidation and AN than the WTO, whereas the RMSE is lower or equal. These results also suggest that this deviation is caused by the relatively small change of oxidation and AN in the data set of the MGO as described in Table 1.

We took repeated readings from some oil samples in order to measure the repeatability independent of the absolute measurement inaccuracy introduced by the calibration model. Averaging over the WTO and the MGO samples, the achieved repeatability (95% confidence range) was 0.19 A/cm for the oxidation and 0.04 mgKOH/g for the AN using the appropriate calibration models for each oil type. Testing the repeatability of the measured water content in the AGO samples with the appropriate calibration model led to a confidence range of 0.23 A/cm or 18.6 ppm.

The results presented in Table 3 and Figure 8 suggest a linear relationship between infrared absorption and oil condition parameters, which is consistent with the Lambert–Beer law. This is particularly noticeable for the data sets in which the condition parameters are evenly distributed over a large value range—namely, the water content of the AGO and the oxidation and AN of the WTO. In future work, water-contaminated WTO and MGO samples as well as MGO samples with increased oxidation and AN should be used to validate the sensor performance over the entire relevant range of these oil condition parameters.

## 7. Summary and Conclusions

In this paper, we presented the development of a sensor for monitoring gearbox lubricants. The sensor can be integrated into the oil circuit of a gearbox in order to monitor the condition of the lubricating oil in use. The system works on the principle of non-dispersive infrared absorption measurement and uses a transmission setup consisting of two thin-film infrared emitters and two four-channel pyroelectric detectors. Seven different optical bandpass filters are used to monitor changes in the infrared absorption spectrum of the oil. To select the optical bandpass filters, a multivariate regression analysis of the used oil infrared absorption spectra was performed.

We performed a ray tracing simulation to analyze and correct the influence of the optical sensor setup on the optical characteristics of the bandpass filters. The sensor prototype was implemented in a rugged aluminum housing to withstand the operating requirements of industrial environments.

Calibration models for predicting the oxidation, water content, and acid number of different gearbox oil types were constructed by means of multivariate partial least squares regression. Cross-validation of the calibration models showed good correlation between the laboratory reference data and the values measured with the presented NDIR sensor. The measurement accuracy could be further improved by using customized optical bandpass filters and a larger set of used oil samples to generate the calibration model.

Compared to oil condition sensors based on impedance spectroscopy [1,2], particle monitoring [3,4], or viscosity measurement [5], the presented NDIR sensor uses the dependence of infrared absorption on the concentration of various chemical compounds to determine the oil condition. In this way, different changes in the chemical composition of the oil can be measured separately, whereby a more detailed determination of the oil condition can be achieved. Compared with NDIR sensors that can determine single condition parameters like oxidation [11] or additive depletion [10], the sensor presented here is capable of measuring three different oil condition parameters simultaneously. Through the use of multivariate PLS regression for the sensor calibration, the information content of all infrared channels is used to determine oxidation, water content, and AN of the oil. The optical bandpass filters for NDIR condition monitoring of phosphate-ester hydraulic fluid [12,13], gas engine oil [14] and diesel engine oil [15] were selected on the basis of previous knowledge of the chemical reactions occurring during the oil aging process. We have shown that the bandpass filters can also be selected by means of a regression analysis, which means that no prior knowledge of the chemical aging behavior of the oil is necessary. This technique could thus easily be applied to select bandpass filters for other oil types or condition parameters.

Because of its quasi-continuous data acquisition, the sensor is able to represent a supplementary tool to complex laboratory analysis for the condition monitoring of lubricating oil in gearbox applications. When integrated into a lubrication circuit, the sensor can immediately detect water ingress, and appropriate measures can be taken to prevent damage to the machinery. Oil change intervals can be adjusted to the actual demand by automatically monitoring the oil oxidation and the acid number over long periods of time, which reduces the operating and service costs.

## Figures and Tables

**Table 1:** Overview of the oil samples at our disposal. The samples of the wind turbine oil (WTO) and the marine gearbox oil (MGO) are aged in real applications, while the samples of the automotive gearbox oil (AGO) consist of fresh oil artificially contaminated with different concentrations of distilled water.

Oil Type	Sample Count	Water Content Range (A/cm)	Oxidation Range (A/cm)	AN Range (mgKOH/g)
WTO	46	6.19–9.62	2.88–7.04	0.38–1.14
MGO	24	5.57–7.15	3.27–4.82	0.12–0.56
AGO	9	8.67–32.7		

**Table 2:** Overview of the oil condition parameters to be monitored and the corresponding optical bandpass filters selected for the implementation of the NDIR sensor. FWHM: full width at half maximum.

Spectral Feature	Target ${\tilde{\nu }}_{\Theta }$ (cm${}^{-1}$)	Target ${\tilde{\nu }}_{⊥}$ (cm${}^{-1}$)	Commercial Filter ${\tilde{\nu }}_{⊥}$/FWHM (cm${}^{-1}$)
Baseline point 1	3900	3881	3876/75
Water content	3409	3393	3390/57
Baseline point 2	2100	2090	2119/40
Oxidation and AN influence	1710	1702	1729/27
AN influence	1606	1598	1608/36
AN influence	1162	1156	1151/27
AN influence	1040	1035	n. a.
AN influence	987	982	980/9
AN influence	722	718	n. a.

**Table 3:** Summary of the model validation results for all oil types and condition parameters.

Oil Type	Indicator	Water Content	Oxidation	AN
	R^2^	0.81	0.95	0.93
WTO	RMSE	0.40	0.29	0.05
	Slope	0.84	0.96	0.94
	Intercept	1.25	0.22	0.04
	R^2^	0.82	0.86	0.77
MGO	RMSE	0.19	0.15	0.05
	Slope	0.84	0.89	0.81
	Intercept	0.99	0.39	0.05
	R^2^	0.99		
AGO	RMSE	0.57		
	Slope	1.00		
	Intercept	0.05		
